# Aqua­bis(2-methyl-4-oxopyrido[1,2-*a*]pyrimidin-9-olato)zinc(II) monohydrate

**DOI:** 10.1107/S1600536808039615

**Published:** 2008-12-17

**Authors:** Yu-Feng Wei, Zhong-Shu Li, Huai-Hong Zhang, Yi-Hong Wang

**Affiliations:** a Ordered Matter Science Research Center, College of Chemistry and Chemical Engineering, Southeast University, Nanjing 210096, People’s Republic of China

## Abstract

The crystal structure of the title compound, [Zn(C_9_H_7_N_2_O_2_)_2_(H_2_O)]·H_2_O, involves discrete mononuclear complex mol­ecules. The special positions on the rotation twofold axis are occupied by Zn^II^ and O atoms of the coordinated and uncoordinated water mol­ecules. The coordination around the Zn^II^ atom can be described as transitional from trigonal-bipyramidal to square-pyramidal. The two chelating 2-methyl-4-oxopyrido[1,2-*a*]pyrimidin-9-olate ligands and the coordin­ated water mol­ecule form the Zn coordination. O—H⋯O hydrogen bonds between the coordinated water mol­ecule and the ligand and between the uncoordinated water mol­ecule and the ligand dominate the crystal packing.

## Related literature

For the design and synthesis of self-assembling systems with organic ligands containing N and O donors, see: Bayot *et al.* (2006 ▶); Chen *et al.* (2007 ▶). For the structures of quinolin-8-ol complexes, see: Wu *et al.* (2006 ▶).
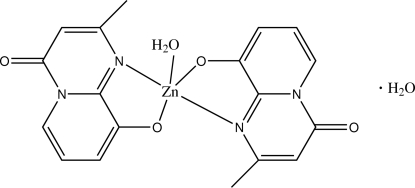


## Experimental

### 

#### Crystal data

[Zn(C_9_H_7_N_2_O_2_)_2_(H_2_O)]·H_2_O
*M* *_r_* = 451.73
Orthorhombic, 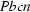

*a* = 7.7670 (16) Å
*b* = 16.045 (3) Å
*c* = 14.006 (3) Å
*V* = 1745.4 (6) Å^3^
*Z* = 4
Mo *K*α radiation
μ = 1.46 mm^−1^
*T* = 293 (2) K
0.25 × 0.15 × 0.12 mm

#### Data collection

Rigaku Scxmini 1K CCD area-detector diffractometer
Absorption correction: multi-scan (*CrystalClear*; Rigaku, 2005 ▶) *T*_min_ = 0.752, *T*_max_ = 0.831
16899 measured reflections
2005 independent reflections
1470 reflections with *I* > 2σ(*I*)
*R*_int_ = 0.070

#### Refinement

*R*[*F* ^2^ > 2σ(*F* ^2^)] = 0.049
*wR*(*F* ^2^) = 0.128
*S* = 1.07
2005 reflections
141 parameters
H atoms treated by a mixture of independent and constrained refinement
Δρ_max_ = 0.50 e Å^−3^
Δρ_min_ = −0.56 e Å^−3^

### 

Data collection: *CrystalClear* (Rigaku, 2005 ▶); cell refinement: *CrystalClear*; data reduction: *CrystalClear*; program(s) used to solve structure: *SHELXS97* (Sheldrick, 2008 ▶); program(s) used to refine structure: *SHELXL97* (Sheldrick, 2008 ▶); molecular graphics: *SHELXTL* (Sheldrick, 2008 ▶); software used to prepare material for publication: *SHELXTL*.

## Supplementary Material

Crystal structure: contains datablocks I, global. DOI: 10.1107/S1600536808039615/kp2191sup1.cif

Structure factors: contains datablocks I. DOI: 10.1107/S1600536808039615/kp2191Isup2.hkl

Additional supplementary materials:  crystallographic information; 3D view; checkCIF report

## Figures and Tables

**Table 1:** Hydrogen-bond geometry (Å, °)

*D*—H⋯*A*	*D*—H	H⋯*A*	*D*⋯*A*	*D*—H⋯*A*
O3—H3*B*⋯O2^i^	0.72 (4)	2.11 (4)	2.823 (3)	170 (5)
O4—H4*B*⋯O1^ii^	0.78 (5)	2.23 (5)	3.008 (4)	176 (6)

Symmetry codes: (i) 
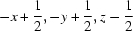
; (ii) 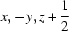.

**Fig. 1.**
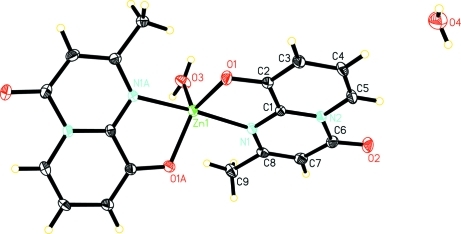
The molecular structure of the title molecule and the atom-numbering scheme. Displacement ellipsoids are drawn at the 30% probability level. [Symmetry code A: -x, y, 0.5 - z.]

**Fig. 2.**
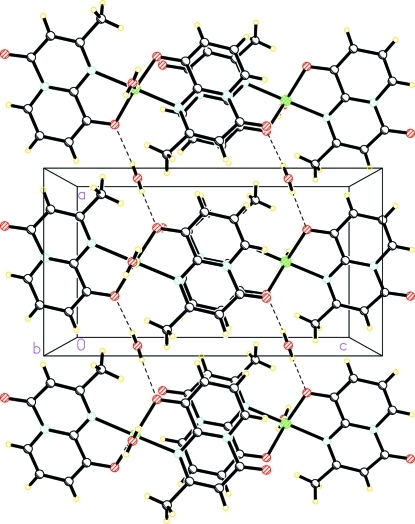
Crystal packing of the compound (I). Hydrogen bonds are shown as dashed lines.

## supplementary crystallographic information

### Comment

Considerable attention has been paid to the design and synthesis of self-assembling systems with organic ligands containing N and O donors (Bayot *et al.*, 2006; Chen, *et al.*, 2007). Quinolin-8-ol is one such ligand and several crystal structures of complexes containing it have been reported (Wu *et al.*, 2006). We report here the synthesis and crystal structure of the title complex, (I) (Fig. 1). In (I), the Zn atom is penta-coordinated by two pyridine nitrogen atoms and two oxygen atoms from the hydroxy groups and water molecule (Fig. 1 and Table 1). Intermolecular O—H···O hydrogen bonds (Table 2 and Fig. 2) connect the molecules of (I) define the crystal packing.

### Experimental

All chemicals used (reagent grade) were commercially available. An aqueous solution (5 ml) of ZnCl_2_ (13.6 mg, 0.1 mmol) was added by constant stirring to an ethanol solution (10 ml) containing 2-methyl-9-hydroxylpyrido [1,2-*a*]pyrimidin-4-one (17.6 mg, 0.1 mmol) then filtered off. After a few days, colourless, well shaped single crystals in the form of prisms deposited in the mother-liquid. They were separated off, washed with cold ethanol and dried in air at room temperature.

### Refinement

In general, H atoms bound to carbon were placed in geometrical positions and refined using a riding model, with C—H = 0.94Å and *U*_iso_(H) = 1.2*U*_eq_(C). The H of water were located from the difference map and refined freely.

### Crystal data

**Table:**

[Zn(C_9_H_7_N_2_O_2_)_2_(H_2_O)]·H_2_O	*F*(000) = 928
*M**_r_* = 451.73	*D*_x_ = 1.719 Mg m^−^^3^
Orthorhombic, *P**b**c**n*	Mo *K*α radiation, λ = 0.71073 Å
Hall symbol: -P 2n 2ab	Cell parameters from 13380 reflections
*a* = 7.7670 (16) Å	θ = 3.0–27.6°
*b* = 16.045 (3) Å	µ = 1.46 mm^−^^1^
*c* = 14.006 (3) Å	*T* = 293 K
*V* = 1745.4 (6) Å^3^	Prism, colourless
*Z* = 4	0.25 × 0.15 × 0.12 mm

### Data collection

**Table:**

Rigaku Scxmini 1K CCD area-detector diffractometer	2005 independent reflections
Radiation source: fine-focus sealed tube	1470 reflections with *I* > 2σ(*I*)
graphite	*R*_int_ = 0.070
Detector resolution: 8.192 pixels mm^-1^	θ_max_ = 27.5°, θ_min_ = 3.3°
Thin–slice ω scans	*h* = −10→10
Absorption correction: multi-scan (*CrystalClear*; Rigaku, 2005)	*k* = −20→20
*T*_min_ = 0.752, *T*_max_ = 0.831	*l* = −18→18
16899 measured reflections	

### Refinement

**Table:**

Refinement on *F*^2^	Primary atom site location: structure-invariant direct methods
Least-squares matrix: full	Secondary atom site location: difference Fourier map
*R*[*F*^2^ > 2σ(*F*^2^)] = 0.049	Hydrogen site location: inferred from neighbouring sites
*wR*(*F*^2^) = 0.128	H atoms treated by a mixture of independent and constrained refinement
*S* = 1.07	*w* = 1/[σ^2^(*F*_o_^2^) + (0.0591*P*)^2^ + 2.2542*P*] where *P* = (*F*_o_^2^ + 2*F*_c_^2^)/3
2005 reflections	(Δ/σ)_max_ < 0.001
141 parameters	Δρ_max_ = 0.50 e Å^−^^3^
0 restraints	Δρ_min_ = −0.56 e Å^−^^3^

### Special details

**Table:**

Geometry. All e.s.d.'s (except the e.s.d. in the dihedral angle between two l.s. planes) are estimated using the full covariance matrix. The cell e.s.d.'s are taken into account individually in the estimation of e.s.d.'s in distances, angles and torsion angles; correlations between e.s.d.'s in cell parameters are only used when they are defined by crystal symmetry. An approximate (isotropic) treatment of cell e.s.d.'s is used for estimating e.s.d.'s involving l.s. planes.
Refinement. Refinement of *F*^2^ against ALL reflections. The weighted *R*-factor *wR* and goodness of fit *S* are based on *F*^2^, conventional *R*-factors *R* are based on *F*, with *F* set to zero for negative *F*^2^. The threshold expression of *F*^2^ > σ(*F*^2^) is used only for calculating *R*-factors(gt) *etc*. and is not relevant to the choice of reflections for refinement. *R*-factors based on *F*^2^ are statistically about twice as large as those based on *F*, and *R*- factors based on ALL data will be even larger.

### Fractional atomic coordinates and isotropic or equivalent isotropic displacement parameters (Å^2^)

**Table:**

	*x*	*y*	*z*	*U*_iso_*/*U*_eq_	
C1	−0.0089 (4)	0.1222 (2)	0.4495 (2)	0.0220 (7)	
C2	−0.1741 (4)	0.0991 (2)	0.4103 (2)	0.0242 (7)	
C3	−0.2932 (5)	0.0659 (2)	0.4714 (3)	0.0290 (8)	
H3A	−0.4012	0.0508	0.4484	0.035*	
C4	−0.2541 (5)	0.0543 (2)	0.5686 (2)	0.0301 (8)	
H4A	−0.3366	0.0317	0.6092	0.036*	
C5	−0.0987 (5)	0.0754 (2)	0.6036 (2)	0.0308 (8)	
H5A	−0.0747	0.0668	0.6680	0.037*	
C6	0.1875 (5)	0.1347 (2)	0.5853 (2)	0.0287 (8)	
C7	0.3026 (5)	0.1688 (2)	0.5193 (3)	0.0301 (8)	
H7A	0.4105	0.1859	0.5402	0.036*	
C8	0.2616 (4)	0.1779 (2)	0.4246 (2)	0.0240 (7)	
C9	0.3867 (5)	0.2129 (2)	0.3541 (3)	0.0332 (9)	
H9A	0.3346	0.2143	0.2920	0.050*	
H9B	0.4877	0.1784	0.3523	0.050*	
H9C	0.4182	0.2684	0.3729	0.050*	
N1	0.1066 (4)	0.15560 (17)	0.39001 (19)	0.0230 (6)	
N2	0.0251 (3)	0.10953 (18)	0.54498 (19)	0.0235 (6)	
O1	−0.1984 (3)	0.11255 (17)	0.31857 (17)	0.0322 (6)	
O2	0.2095 (4)	0.12461 (18)	0.67183 (17)	0.0383 (7)	
Zn1	0.0000	0.16492 (4)	0.2500	0.0259 (2)	
O3	0.0000	0.2946 (3)	0.2500	0.0420 (10)	
O4	−0.5000	−0.0113 (3)	0.7500	0.0529 (12)	
H4B	−0.420 (7)	−0.038 (4)	0.765 (4)	0.08 (2)*	
H3B	0.069 (6)	0.320 (3)	0.232 (3)	0.046 (15)*	

### Atomic displacement parameters (Å^2^)

**Table:**

	*U*^11^	*U*^22^	*U*^33^	*U*^12^	*U*^13^	*U*^23^
C1	0.0236 (16)	0.0234 (15)	0.0189 (15)	0.0037 (15)	0.0017 (14)	−0.0002 (12)
C2	0.0215 (17)	0.0284 (18)	0.0225 (17)	0.0009 (14)	0.0003 (14)	0.0009 (14)
C3	0.0224 (17)	0.037 (2)	0.0275 (18)	−0.0033 (15)	0.0014 (15)	0.0022 (15)
C4	0.0279 (19)	0.037 (2)	0.0254 (18)	−0.0065 (16)	0.0099 (15)	0.0032 (16)
C5	0.035 (2)	0.038 (2)	0.0197 (16)	−0.0017 (17)	0.0052 (16)	0.0021 (15)
C6	0.0291 (19)	0.0329 (19)	0.0241 (17)	0.0021 (16)	−0.0055 (15)	−0.0049 (15)
C7	0.0211 (17)	0.036 (2)	0.0335 (18)	−0.0014 (15)	−0.0031 (15)	−0.0063 (16)
C8	0.0206 (16)	0.0242 (17)	0.0273 (17)	0.0008 (13)	0.0030 (14)	−0.0064 (14)
C9	0.030 (2)	0.040 (2)	0.0295 (18)	−0.0102 (17)	0.0041 (16)	−0.0066 (16)
N1	0.0222 (15)	0.0285 (15)	0.0185 (13)	−0.0024 (12)	0.0024 (11)	−0.0012 (11)
N2	0.0234 (16)	0.0295 (15)	0.0177 (13)	0.0005 (12)	0.0015 (11)	−0.0008 (11)
O1	0.0234 (13)	0.0505 (16)	0.0228 (12)	−0.0071 (12)	−0.0026 (10)	0.0072 (11)
O2	0.0387 (16)	0.0545 (17)	0.0218 (13)	−0.0045 (14)	−0.0078 (11)	0.0000 (12)
Zn1	0.0240 (3)	0.0347 (3)	0.0191 (3)	0.000	0.0024 (2)	0.000
O3	0.040 (2)	0.032 (2)	0.054 (3)	0.000	0.021 (2)	0.000
O4	0.046 (3)	0.059 (3)	0.054 (3)	0.000	0.000 (3)	0.000

### Geometric parameters (Å, °)

**Table:**

C1—N1	1.336 (4)	C7—C8	1.372 (5)
C1—N2	1.378 (4)	C7—H7A	0.9300
C1—C2	1.444 (5)	C8—N1	1.346 (4)
C2—O1	1.316 (4)	C8—C9	1.494 (5)
C2—C3	1.368 (5)	C9—H9A	0.9600
C3—C4	1.408 (5)	C9—H9B	0.9600
C3—H3A	0.9300	C9—H9C	0.9600
C4—C5	1.346 (5)	N1—Zn1	2.134 (3)
C4—H4A	0.9300	O1—Zn1	2.001 (2)
C5—N2	1.379 (4)	Zn1—O1^i^	2.001 (2)
C5—H5A	0.9300	Zn1—O3	2.081 (4)
C6—O2	1.234 (4)	Zn1—N1^i^	2.134 (3)
C6—C7	1.398 (5)	O3—H3B	0.72 (4)
C6—N2	1.440 (4)	O4—H4B	0.78 (5)
			
N1—C1—N2	122.4 (3)	C8—C9—H9A	109.5
N1—C1—C2	117.5 (3)	C8—C9—H9B	109.5
N2—C1—C2	120.1 (3)	H9A—C9—H9B	109.5
O1—C2—C3	125.2 (3)	C8—C9—H9C	109.5
O1—C2—C1	117.2 (3)	H9A—C9—H9C	109.5
C3—C2—C1	117.6 (3)	H9B—C9—H9C	109.5
C2—C3—C4	120.7 (3)	C1—N1—C8	118.9 (3)
C2—C3—H3A	119.7	C1—N1—Zn1	109.9 (2)
C4—C3—H3A	119.7	C8—N1—Zn1	131.2 (2)
C5—C4—C3	120.9 (3)	C1—N2—C5	120.2 (3)
C5—C4—H4A	119.6	C1—N2—C6	120.5 (3)
C3—C4—H4A	119.6	C5—N2—C6	119.3 (3)
C4—C5—N2	120.5 (3)	C2—O1—Zn1	115.3 (2)
C4—C5—H5A	119.7	O1—Zn1—O1^i^	130.33 (16)
N2—C5—H5A	119.7	O1—Zn1—O3	114.83 (8)
O2—C6—C7	127.8 (4)	O1^i^—Zn1—O3	114.83 (8)
O2—C6—N2	118.0 (3)	O1—Zn1—N1^i^	96.48 (10)
C7—C6—N2	114.2 (3)	O1^i^—Zn1—N1^i^	80.11 (10)
C8—C7—C6	122.2 (3)	O3—Zn1—N1^i^	94.02 (7)
C8—C7—H7A	118.9	O1—Zn1—N1	80.11 (10)
C6—C7—H7A	118.9	O1^i^—Zn1—N1	96.48 (10)
N1—C8—C7	121.8 (3)	O3—Zn1—N1	94.02 (7)
N1—C8—C9	116.4 (3)	N1^i^—Zn1—N1	171.96 (15)
C7—C8—C9	121.8 (3)	Zn1—O3—H3B	125 (4)
			
N1—C1—C2—O1	0.1 (5)	N1—C1—N2—C6	2.0 (5)
N2—C1—C2—O1	−179.8 (3)	C2—C1—N2—C6	−178.2 (3)
N1—C1—C2—C3	−179.2 (3)	C4—C5—N2—C1	−0.2 (5)
N2—C1—C2—C3	0.9 (5)	C4—C5—N2—C6	177.5 (3)
O1—C2—C3—C4	−179.8 (3)	O2—C6—N2—C1	177.5 (3)
C1—C2—C3—C4	−0.6 (5)	C7—C6—N2—C1	−2.0 (5)
C2—C3—C4—C5	−0.1 (6)	O2—C6—N2—C5	−0.1 (5)
C3—C4—C5—N2	0.5 (6)	C7—C6—N2—C5	−179.7 (3)
O2—C6—C7—C8	−178.9 (4)	C3—C2—O1—Zn1	178.4 (3)
N2—C6—C7—C8	0.6 (5)	C1—C2—O1—Zn1	−0.9 (4)
C6—C7—C8—N1	1.1 (5)	C2—O1—Zn1—O1^i^	91.1 (2)
C6—C7—C8—C9	−178.7 (3)	C2—O1—Zn1—O3	−88.9 (2)
N2—C1—N1—C8	−0.2 (5)	C2—O1—Zn1—N1^i^	173.6 (2)
C2—C1—N1—C8	179.9 (3)	C2—O1—Zn1—N1	0.9 (2)
N2—C1—N1—Zn1	−179.5 (2)	C1—N1—Zn1—O1	−0.8 (2)
C2—C1—N1—Zn1	0.7 (4)	C8—N1—Zn1—O1	−179.9 (3)
C7—C8—N1—C1	−1.3 (5)	C1—N1—Zn1—O1^i^	−130.7 (2)
C9—C8—N1—C1	178.5 (3)	C8—N1—Zn1—O1^i^	50.2 (3)
C7—C8—N1—Zn1	177.8 (2)	C1—N1—Zn1—O3	113.7 (2)
C9—C8—N1—Zn1	−2.4 (4)	C8—N1—Zn1—O3	−65.4 (3)
N1—C1—N2—C5	179.6 (3)	C1—N1—Zn1—N1^i^	−66.3 (2)
C2—C1—N2—C5	−0.6 (5)	C8—N1—Zn1—N1^i^	114.6 (3)

Symmetry codes: (i) −*x*, *y*, −*z*+1/2.

### Hydrogen-bond geometry (Å, °)

**Table:**

*D*—H···*A*	*D*—H	H···*A*	*D*···*A*	*D*—H···*A*
O3—H3B···O2^ii^	0.72 (4)	2.11 (4)	2.823 (3)	170 (5)
O4—H4B···O1^iii^	0.78 (5)	2.23 (5)	3.008 (4)	176 (6)

Symmetry codes: (ii) −*x*+1/2, −*y*+1/2, *z*−1/2; (iii) *x*, −*y*, *z*+1/2.
